# Psychotherapy training in psychiatry: current opportunities and perspectives of early career psychiatrists

**DOI:** 10.1192/j.eurpsy.2026.10308

**Published:** 2026-07-31

**Authors:** S. Tanyeri Kayahan

**Affiliations:** Psychiatry, Republic of Türkiye Ministry of Health Yalvaç State Hospital, Isparta, Türkiye

## Abstract

**Abstract:**

Psychotherapy is crucial in mental healthcare, and clinical guidelines highlight the need for trainees to acquire core psychotherapeutic skills. Although many programs specify minimum training requirements, implementation varies widely due to resource constraints. In our recent systematic review, we examined early-career psychiatrists’ (ECPs) global perspectives on psychotherapy training (PT) and the key strengths and challenges of training opportunities. We conducted a systematic search of MEDLINE, Scopus, and PubPsych to identify survey-based studies investigating ECPs’ perspectives on PT, following the PRISMA framework. From an initial pool of 31,281 records, 56 studies met the inclusion criteria. Results were analyzed thematically and synthesized narratively. Studies were from Europe (50%), the Americas (26.7%), the Western Pacific (12.5%), South-East Asia (7.2%), the Eastern Mediterranean (1.8%), and Africa (1.8%), with a total of 8,305 participants. Thirty-one studies on ECPs’ interest in PT found that 57–80% were interested in psychotherapy and that 67–92% viewed being a psychotherapist as part of their identity as a psychiatrist. Training opportunities varied across countries and institutions, with cognitive behavioral therapy and psychodynamic psychotherapy as the primary modalities. Strengths included available supervision, structured rotations, and integrative approaches combining multiple modalities. The most frequent challenges were inadequate supervision, insufficient protected time, and limited exposure to diverse modalities, as well as a shortage of trained supervisors and variability in training delivery. Recommendations underscored the need for coherent curricula, stronger supervision, greater institutional support, and improved access to training resources. Improving access to evidence-based, culturally adapted PT is essential, and training institutions and professional organizations play a key role in supporting ongoing professional development.

**Disclosure of Interest:**

None Declared

